# Impressions of Current Clinical Relevance of Gastrointestinal Histopathobiome in Psychiatry

**DOI:** 10.1192/bjo.2026.11607

**Published:** 2026-06-30

**Authors:** Megan Paterson

**Affiliations:** NHS, Ayrshire & Arran, United Kingdom

## Abstract

**Aims:**

The gastrointestinal histopathobiome has major potential clinical implications for Psychiatry. However, the complexity and relatively nascent nature of this research means that its direct clinical relevance is presently unclear. The aim of this study was to obtain a snapshot of current attitudes towards this field amongst psychiatric medical staff.

**Methods:**

Through an anonymous survey sent to psychiatric medical staff working within Ayrshire and Arran in February 2026, contemporary, qualitative data were gathered across four domains:

1. Current medical grade of the doctor

2. To what extent the doctors were likely to specifically seek out information pertaining to the patient’s gastrointestinal tract.

3. To what extent the doctors were likely to incorporate knowledge relating to the histopathobiome into their management plans.

4. The perceived relevance of, and interest in, this field.

Likert scales were used to collect responses, apart from the final question which required either a “yes” or “no”.

**Results:**

Seven responses were obtained, representing the views of three consultants, one registrar, one specialty doctor and two core trainees. FY2s and clinical fellows did not respond.

57% were “very unlikely” to enquire about the gastrointestinal system, and 57% were “unlikely” or “very unlikely” to ask about diet. Results were evenly spread with regard to seeking out gastrointestinal investigations or histology reports.

57% reported having, on occasion, either offered advice or referred to dietetics, specifically to improve the gut microbiome, however do not often feel this is relevant. 86% reported that they would either “probably” or “definitely not” feel comfortable taking a history or advising in this regard.

29% felt this research is currently clinically relevant in Psychiatry, compared with 71% who did not (43% felt it might be in the future but that it is not at present). 86% would be interested in learning more about this topic.

There were no emerging patterns across grade, however, the small sample size is of relevance here.

**Conclusion:**

Findings suggest that this emerging research is not yet deemed clinically useful by Psychiatric staff within the health board. However, there is an awareness of it, and are adiness to learn more. Given the lack of current best practice regarding the gastrointestinal histopathobiome in psychiatric management, the findings are likely appropriate.

